# The Influence of Doping with Transition Metal Ions on the Structure and Magnetic Properties of Zinc Oxide Thin Films

**DOI:** 10.1155/2014/265969

**Published:** 2014-02-10

**Authors:** Jenica Neamtu, Marius Volmer

**Affiliations:** ^1^National Institute for Research and Development in Electrical Engineering, Splaiul Unirii No. 313, Bucharest, Romania; ^2^Transilvania University of Brasov, Eroilor No. 29, Brasov, Romania

## Abstract

Zn_1−*x*_Ni_*x*_O (*x* = 0.03 ÷ 0.10) and Zn_1−*x*_Fe_*x*_O (*x* = 0.03 ÷ 0.15) thin films were synthesized by sol-gel method. The structure and the surface morphology of zinc oxide thin films doped with transition metal (TM) ions have been investigated by X-ray diffraction (XRD) and atomic force microscopy (AFM). The magnetic studies were done using vibrating sample magnetometer (VSM) at room temperature. Experimental results revealed that the substitution of Ni ions in ZnO wurtzite lattice for the contents *x* = 0.03 ÷ 0.10 (Ni^2+^) leads to weak ferromagnetism of thin films. For Zn_1−*x*_Fe_*x*_O with *x* = 0.03 ÷ 0.05, the Fe^3+^ ions are magnetic coupling by superexchange interaction via oxygen ions in wurtzite structure. For *x* = 0.10 ÷ 0.15 (Fe^3+^) one can observe the increasing of secondary phase of ZnFe_2_O_4_ spinel. The Zn_0.9_Fe_0.1_O film shows a superparamagnetic behavior due to small crystallite sizes and the net spin magnetic moments arisen from the interaction between the iron ions through an oxygen ion in the spinel structure.

## 1. Introduction

Since the study of III–V semiconductors doped with transition metals by Ohno [1, 2], many researches were conducted to obtain the room temperature ferromagnetism of diluted magnetic semiconductors (DMS). Ferromagnetism in transition metal (TM)-doped ZnO is theoretically investigated by Sato and Katayama-Yoshida [3] using ab initio calculations based on local density approximation (LDA). The ferromagnetism of TM-doped ZnO is considered through a double-exchange mechanism, without requiring additional carrier incorporation. A few years ago, it turned out that most of incomplete 3d shell metal ions can be used to produce room temperature magnetism in ZnO doped with transition metal (Cu, Mn, Fe, Co, or Ni) [4–9]. It is a great interest because the DMS can be integrated for fabricating transparent spin-based devices [10]. Measurable ferromagnetism at room temperature was reported in cobalt-doped zinc oxide thin films [5] and Co-Mn doped zinc oxide [6]. Films consisting of Zn_1−*x*_Fe_*x*_O were prepared by alternating-target laser ablation deposition, with Fe doping levels ranged from *x* = 0.016 to 0.125 [8]. Also by pulsed laser deposition Ni-doped ZnO thin films were prepared with room temperature ferromagnetism [9].

However, the origin of this ferromagnetism is controversial. The studies on the origin of room temperature ferromagnetism in TM:ZnO films have been connected to substituting positions of TM ions in the ZnO lattice [11]. Further studies evidenced the origin of ferromagnetism as being TM precipitates [6] or clusters embedded in ZnO [12]. The room-temperature ferromagnetism of Co-doped ZnO is correlated with structural defects [13] or by incorporation of donor defects enhancing the ferromagnetic properties of Co:ZnO [14]. On the other hand, various chemical methods, as chemical precipitation, hydrothermal reaction, and sol-gel synthesis, have been developed to prepare nanoparticles and nanowires of zinc oxide doped with transition metal ions [15–17]. However, it is a great challenge to synthesize ZnO thin films doped with the transition metal ions using a simple process, with a low cost, as sol-gel preparation.

The goal of this work is the study of structural and magnetic properties of Ni-doped ZnO (Zn_1−*x*_Ni_*x*_O, *x* = 0.03 ÷ 0.10) and Fe-doped ZnO (Zn_1−*x*_Fe_*x*_O, *x* = 0.03 ÷ 0.15) thin films, both synthesized by sol-gel method. X-ray diffraction (XRD) and atomic force microscopy (AFM) were used to characterize their structure and the surface morphology. The magnetic studies were done using vibrating sample magnetometer (VSM) at room temperature. The VSM results revealed that the contents of *x* = 0.03 ÷ 0.10 (Ni^2+^) lead to weak ferromagnetism of thin films. The Zn_1−*x*_Fe_*x*_O thin films with *x* = 0.03 ÷ 0.05 show a weak ferromagnetism, for *x* = 0.10 Fe^3+^ hysteresis loop of thin films shows a superparamagnetic behavior. The structure and surface morphology of thin films were correlated with magnetic properties of TM:ZnO thin films.

## 2. Materials and Methods

### 2.1. Materials

Zn_1−*x*_Ni_*x*_O (*x* = 0.03; 0.05; 0.10) and Zn_1−*x*_Fe_*x*_O (*x* = 0.03; 0.05; 0.10; 0.15) thin films were synthesized by a sol-gel method. Stoichiometric amounts of zinc acetate-2-hydrate [Zn(CH_3_COO)_2_·2H_2_O] (Fluka 99.5%) and nickel nitrate Ni(NO_3_)_2_·6H_2_O (Aldrich 98%) or, respectively, Fe(NO_3_)_3_·9H_2_O (Aldrich 98%) were each dissolved in 20 mL propanol (C_3_H_8_O) by magnetic stirring at room temperature. Both homogeneous solutions of zinc acetate and nickel nitrate (or iron nitrate) were mixed together and then ethanolamine (NH_2_CH_2_CH_2_OH) (Merk 99.5%) was added drop by drop under vigorous stirring. The resulting solution was then refluxed at 80°C for 4 h, until the solution was converted in a gel.

### 2.2. Preparation of Zinc Oxide Thin Films

The Zn_1−*x*_Ni_*x*_O and Zn_1−*x*_Fe_*x*_O thin films have been deposited from the gels by spin coating method (1500 RPM, 30 seconds) on Si (100) and Crown glass substrates. This procedure was repeated four times. The preheated temperature for film stabilization after each layer deposition was 200°C/20 min. The final films have been calcined at 700°C in air, during 2 hours. The same sol-gel method was used for preparation of ZnO thin film, starting to the zinc acetate Zn(CH_3_COO)_2_·2H_2_O (Fluka 99.5%), propanol, and ethanolamine (NH_2_CH_2_CH_2_OH). The same procedure was used for the deposition of ZnO thin films on Si (100) and Crown glass substrates and final treatment at 700°C in air.

### 2.3. Characterization

The thickness of the ZnO thin films, the Zn_1−*x*_Ni_*x*_O and Zn_1−*x*_Fe_*x*_O thin films, measured using a FILMETRICS F20 thin film analyzer, were ranged between 70 and 90 nm.

X-ray diffraction (XRD) of the films was carried out using Bruker-AXS type D8 ADVANCE X-ray diffractometer with Cu-k_*β*_ radiation of 1.5406 $\overset{´}{Å}$, at a step of 0.04°/s in the range 2*θ* = 10°–100°. Surface morphology and roughness of the samples were investigated by atomic force microscopy using “Ntegra aura” microscope with NSG tip (10 nm resolution).

The characterization of magnetic properties at room temperature is done using a LAKESHORE 7300 vibrating sample magnetometer (VSM).

## 3. Results and Discussion

**Figure 1:**
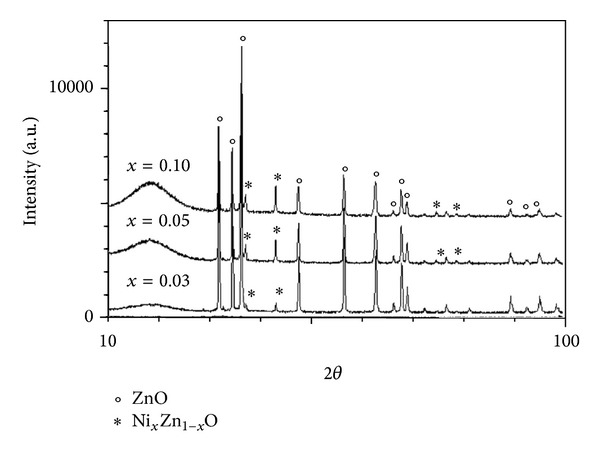
X-ray diffraction patterns of Zn_1−*x*_Ni_*x*_O (*x* = 0.03 ÷ 0.10) thin film samples.

Figure 1 shows the XRD diffraction patterns of nickel doped zinc oxide (Zn_1−*x*_Ni_*x*_O, where *x* = 0.03 ÷ 0.10) thin films, sintered at 700°C temperature, for 2 h, in air. From X-ray diffraction intensity distribution it is observed that the peaks of wurtzite structure are majorities, indicating that these thin films have a structure similar to ZnO, in agreement with the reported JCPDS card no. 36-1451 ($a=b=3.249\;\overset{´}{Å}$ and $c=5.206\;\overset{´}{Å}$). One can conclude that Ni^2+^ ions occupy the Zn^2+^ sites into the crystal lattice of ZnO. As can be seen in this figure with the increase of Ni^2+^  ions content the diffraction peaks arise for a secondary phase of rhombohedral Zn_1−*x*_Ni_*x*_O, 2*θ* = 37° and 43° and lattice parameters: $a=b=2.962\;\overset{´}{Å}$, $c=7.24\;\overset{´}{Å}$, and $V=55.10\;{\overset{´}{Å}}^{3}$ [18].

Table 1 shows that the lattice parameters of Zn_1−*x*_Ni_*x*_O (*x* = 0.03 ÷ 0.1) are slightly smaller than those of pure ZnO, because of small difference between the ionic radius of the elements ($r{\text{Zn}}^{2+}=0.60\;\overset{´}{Å}$ and $r{\text{Ni}}^{2+}=0.55\;\overset{´}{Å}$ in tetrahedral coordination). The mean crystalline size, calculated from the full-width at half maximum (FWHM) of XRD lines by using the Debye-Scherrer formula [18], increases from 30.9 nm to 47.3 nm with the increased concentration of Ni_*x*_ (*x* = 0.03 ÷ 0.10).

**Figure 2:**
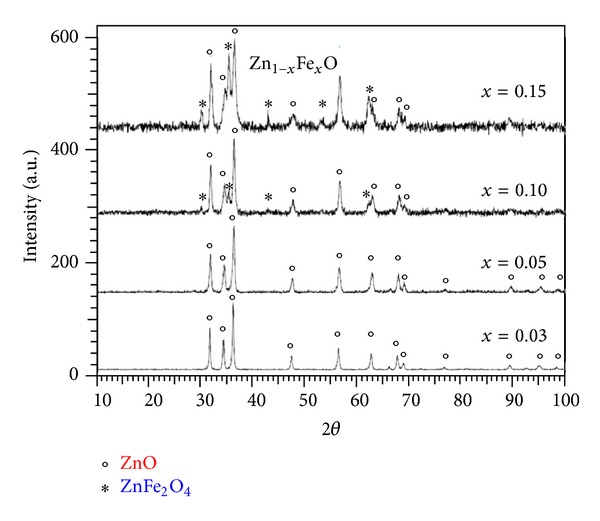
X-ray diffraction patterns of Zn_1−*x*_Fe_*x*_O (*x* = 0.03 ÷ 0.15) thin film samples.

Figure 2 shows the XRD diffraction patterns of iron doped zinc oxide (Zn_1−*x*_Fe_*x*_O, where *x* = 0.03 ÷ 0.15) thin films, sintered at a temperature of 700°C, for 2 h, in air. One can observe that the peaks of wurtzite structure ($a=b=3.249\;\overset{´}{Å}$ and $c=5.206\;\overset{´}{Å}$) are majorities.

From the XRD diffraction patterns of Zn_1−*x*_Fe_*x*_O (where *x* = 0.10 ÷ 0.15) one can observe the peaks of secondary cubic phase at 2*θ* = 29.9°, 35.7°, 43°, and 61.5°. This phase, ZnFe_2_O_4_, is a normal spinel with tetrahedral (A) sites occupied by Zn^2+^ ions and octahedral (B) sites occupied by Fe^3+^ and Fe^2+^ ions [19]. In spinel structure Fe^3+^ has ionic radius 0.55 $\overset{´}{Å}$ (in octahedral coordination) matching that of Zn^2+^ (0.6 Å in tetrahedral coordination) which occupy the tetrahedral holes. Recent studies of XPS spectra indicated a very small amount of Zn in the B-site [20].

Table 2 shows the lattice parameters of Zn_1−*x*_Fe_*x*_O (*x* = 0.03 ÷ 0.15) comparatively with the lattice parameters of pure ZnO and the mean crystalline size, calculated from FWHM of XRD lines by using the Debye-Scherrer formula [18].

From Tables 1 and 2 one can notice that the lattice parameters of doped Zn_1−*x*_TM_*x*_O are close of ZnO wurtzite lattice parameters. The good compromise between the ionic radius matches that of zinc (0.60 Å) versus Ni^2+^ (0.55 Å) and zinc (0.60 Å) versus Fe^3+^ (0.49 Å) both in tetrahedral coordination and it is followed of substitution of Zn^2+^ with Ni^2+^ and Fe^3+^ ions.

**Figure 3:**
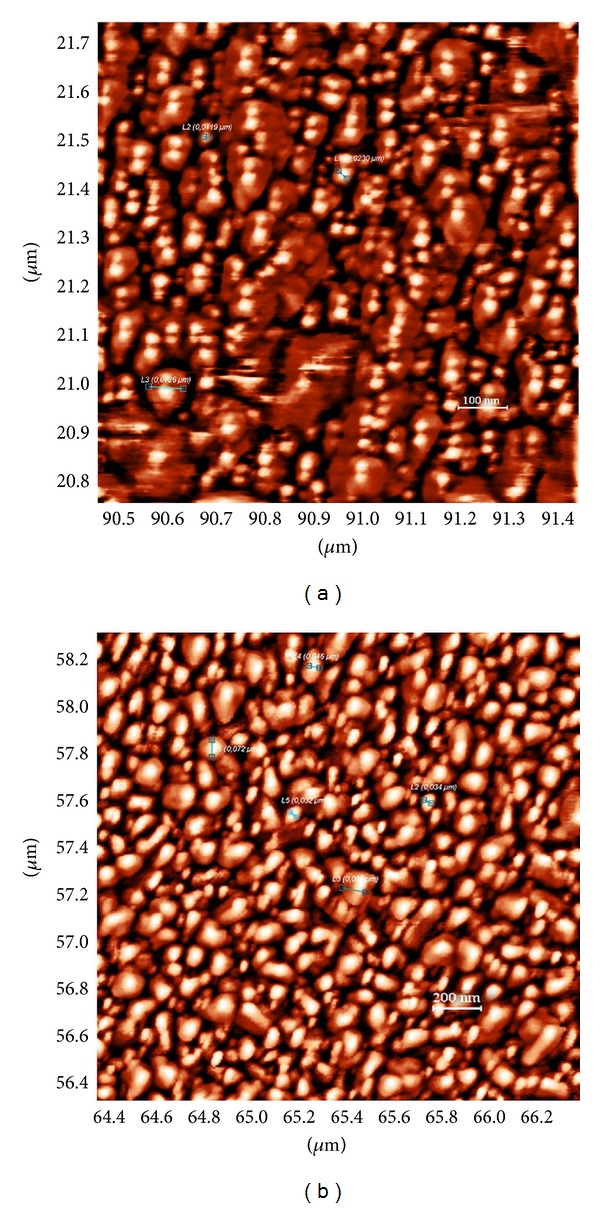
AFM micrographs of thin film surface for (a) Zn_0.97_Ni_0.03_O and (b) Zn_0.90_Ni_0.10_O.

Figure 3(a) shows the morphology of Zn_0.97_Ni_0.03_O thin film and Figure 3(b) shows the morphology of Zn_0.90_Ni_0.10_O thin film, both deposited on Si (100) substrate.

Average crystallite size increases from 35 nm for Zn_0.97_Ni_0.03_O thin film to 56 nm for Zn_0.90_Ni_0.10_O thin film. This tendency of increase is in good accordance with the observed increase of mean crystalline size with the Ni_*x*_ concentration, calculated from the FWHM of XRD lines by using the Debye-Scherrer formula (Table 1).

**Figure 4:**
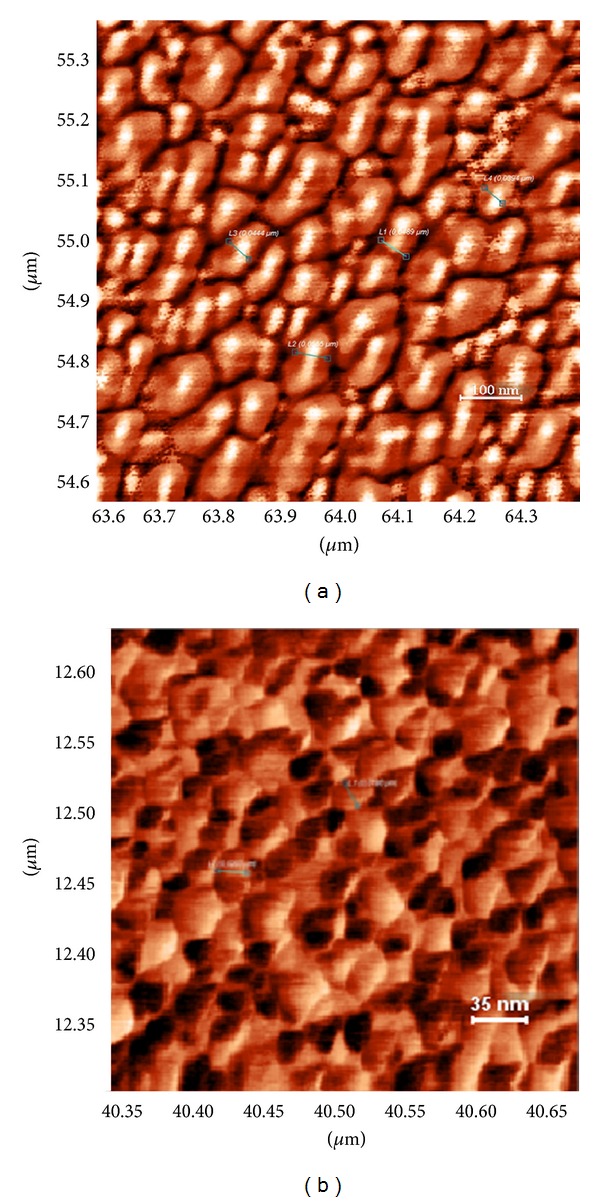
AFM micrographs of thin film surface for (a) Zn_0.97_Fe_0.03_O and (b) Zn_0.90_Fe_0.10_O.

Figure 4(a) shows the morphology of Zn_0.97_Fe_0.03_O thin film and Figure 4(b) shows the morphology of Zn_0.90_Fe_0.10_O thin film, both deposited on Si (100) substrate.

Average crystallite size decreases from 46 nm for Zn_0.97_Fe_0.03_O thin film to 19.5 nm for Zn_0.90_Fe_0.10_O thin film. This tendency of decrease is similar to the observed decrease of mean crystalline size with the Fe_*x*_ concentration, calculated from the FWHM of XRD lines by using the Debye-Scherrer formula (Table 2).

From AFM characterization it is found that all analyzed thin films are formed by close package of crystallites with the holes. It is found that the volume of holes decreases for Zn_1−*x*_Fe_*x*_O by comparison with Zn_1−*x*_Ni_*x*_O thin films.

**Figure 5:**
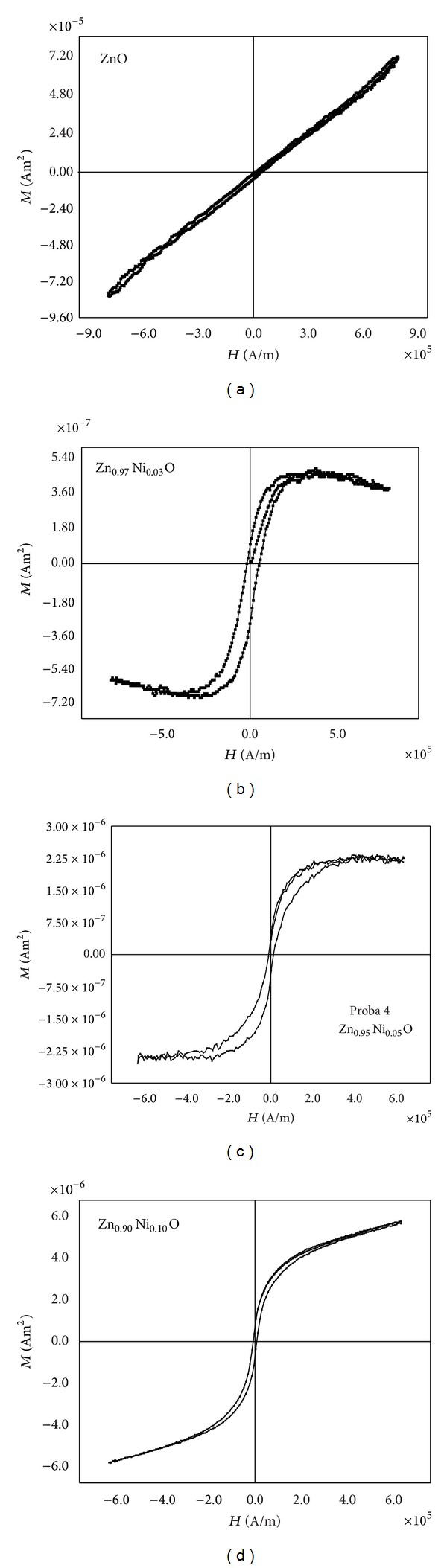
Magnetic hysteresis curves of (a) ZnO, (b) Zn_0.97_Ni_0.03_O, (c) Zn_0.95_Ni_0.05_O, and (d) Zn_0.90_Ni_0.10_O thin films.

Figure 5 shows the magnetization versus the magnetic field measured at room temperature by vibrating sample magnetometer (VSM) for (a) ZnO, (b) Zn_0.97_Ni_0.03_O, (c) Zn_0.95_Ni_0.05_O, and (d) Zn_0.90_Ni_0.10_O thin film samples. The experimental values are not corrected with respect to the diamagnetic contribution of the substrate. Comparatively with undoped ZnO film, all Ni-doped ZnO films show distinctly hysteresis loops, indicating that samples have room-temperature ferromagnetism. The magnetic moments increase with the content of Ni ions from *M*_*s*_ = 5 × 10^−7^ Am^2^ (for *x* = 0.03), *M*_*s*_ = 2.4 × 10^−6^ Am^2^ (for *x* = 0.05), and *M*_*s*_ = 6 × 10^−6^ Am^2^ (for *x* = 0.1). The coercivity (*H*_*c*_) shows decreasing values with the content of Ni ions: *H*_*c*_ = 5 × 10^4^ A/m (for *x* = 0.03), *H*_*c*_ = 2 × 10^4^ A/m (for *x* = 0.05), and *H*_*c*_ = 1 × 10^4^ A/m (for *x* = 0.1). The sample Zn_0.90_Ni_0.10_O is magnetically unsaturated at maximum magnetic field. This observation can be analyzed in terms of change in lattice spacing from the secondary rhombohaedral phase and in terms of superexchange interaction.

**Figure 6:**
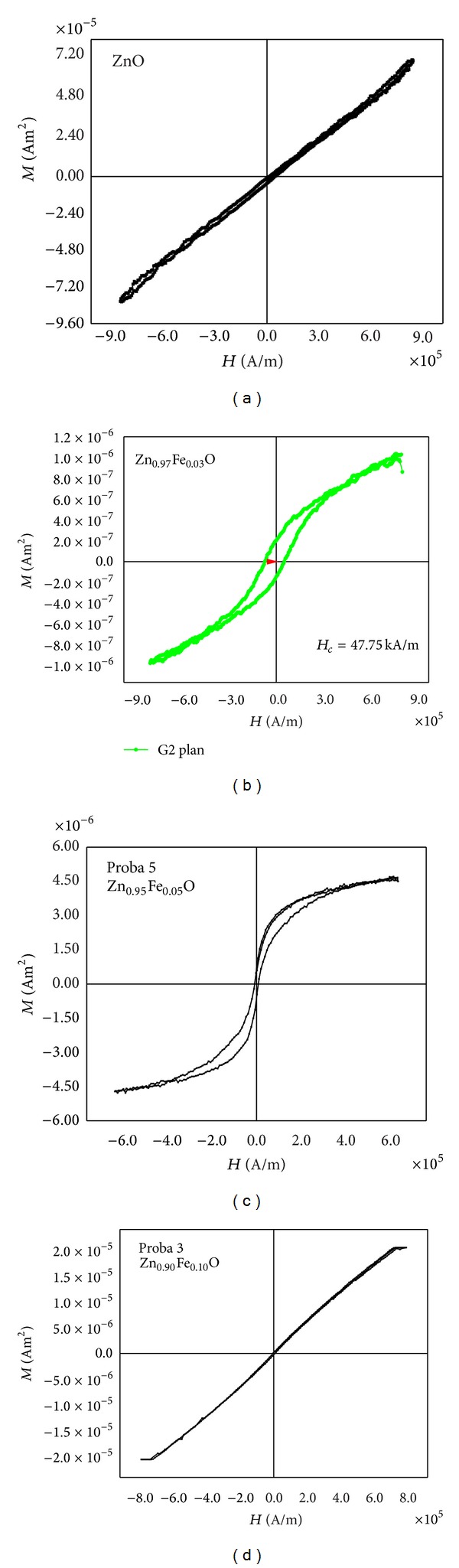
Magnetic hysteresis curves of (a) ZnO, (b) Zn_0.97_Fe_0.03_O, (c) Zn_0.95_Fe_0.05_O, and (d) Zn_0.90_Fe_0.10_O thin films.

Figure 6 shows the magnetization versus the magnetic field measured at room temperature by VSM for (a) ZnO (b) Zn_0.97_Fe_0.03_O, (c) Zn_0.95_Fe_0.05_O, and (d) Zn_0.90_Fe_0.10_O thin films.

The experimental values are not corrected with respect to the diamagnetic contribution of the substrate. By comparison with undoped ZnO film, the Fe-doped ZnO films show hysteresis loops, indicating the room-temperature ferromagnetism of thin films. The magnetic moments increase with the amount of Fe ions from *M*_*s*_ = 1 × 10^−6^ Am^2^ (for *x* = 0.03), *M*_*s*_ = 5 × 10^−6^ Am^2^ (for *x* = 0.05), and *M*_*s*_ = 2 × 10^−5^ Am^2^ (for *x* = 0.1). The coercivity (*H*_*c*_) shows decreasing values with the content of Fe ions, *H*_*c*_ = 5 × 10^4^ A/m (for *x* = 0.03), *H*_*c*_ = 1 × 10^4^ A/m (for  *x* = 0.05), and *H*_*c*_ = 1 × 10^2^ A/m (for *x* = 0.1).

A question can arise from superparamagnetic “hysteresis” behavior of Zn_0.90_Fe_0.10_O thin films. This observation can be explained in terms of small crystallite size (approximately 20 nm from AFM visualization of grains) and in terms of superexchange interaction between Fe^3+^ and Fe^2+^ ions in octahedral (B) sites of ZnFe_2_O_4_ spinel phase.

## 4. Conclusions

Ni-doped ZnO (Zn_1−*x*_Ni_*x*_O, *x* = 0.03 ÷ 0.10) and Fe-doped ZnO (Zn_1−*x*_Fe_*x*_O, *x* = 0.03 ÷ 0.15) thin films have been synthesized using a simple sol-gel method. The results from the structural AFM and magnetic characterization reveal that the levels of doping ions are crucial for obtaining the magnetic properties at room temperature. The XRD analysis shows ZnO wurtzite structure for the Zn_1−*x*_Ni_*x*_O thin films. A possible mechanism for room-temperature ferromagnetism of Zn_1−*x*_Ni_*x*_O is magnetic coupling between Ni^2+^ ions by superexchange interaction via O^2−^ in wurtzite structure. The observation that Zn_0.90_Ni_0.10_O film is magnetically unsaturated, at maximum magnetic field, can be interpreted in terms of change in lattice spacing due to the secondary rhombohedral phase of Zn_1−*x*_Ni_*x*_O, and in terms of superexchange interaction.

From XRD diffraction patterns of iron doped zinc oxide one can observe that the peaks of wurtzite structure are majorities. For Zn_1−*x*_Fe_*x*_O samples, where *x* = 0.03 ÷ 0.05, a magnetic coupling takes place between Fe^3+^ ions by superexchange interaction via oxygen atoms.

The Zn_0.9_Fe_0.1_O film shows a superparamagnetic behavior due to small crystallite sizes and a superexchange interaction between the resultant Fe_*x*_^3+^ and original Fe_*x*_^2+^ ions through an oxygen ion in the B-site. As reported in a number of established papers [21, 22] in the B-site, however, the difference between Fe^3+^ and Fe^2+^ ions cannot be recognized and the spin directions in Fe^3+^ and Fe^2+^ ions are the same, by double-exchange interaction leading to electron hopping between vicinal Fe^3+^ and Fe^2+^ ions through a hybrid orbital formed with the 2p orbital of an oxygen ion.

Our experimental studies confirm that the origin of room temperature ferromagnetism in TM:ZnO films can be connected to substituting positions of TM ions in the ZnO lattice.

## Figures and Tables

**Table 1:** Lattice parameters calculated from the XRD data of Zn_1−*x*_Ni_*x*_O (*x* = 0.03 ÷ 0.10) and mean crystalline size (with Debye-Scherrer formula [18]).

Zn_1−*x*_Ni_*x*_O (*x* = 0.03 ÷ 0.10)			
*x* = 0	*x* = 0.03	*x* = 0.05	*x* = 0.10
$\left[\overset{´}{Å}\right]$ *a* = *b* = 3.249	*a* = *b* = 3.239	*a* = *b* = 3.230	*a* = *b* = 3.236
$[\overset{´}{Å}]\;\;c=5.206$	*c* = 5.189	*c* = 5.193	*c* = 5.192
$\left[{\overset{´}{Å}}^{3}\right]$ *V* = 47.590	*V* = 47.143	*V* = 46.918	*V* = 47.237

[nm] average size	*D* = 30.9	*D* = 32.8	*D* = 47.3

**Table 2:** Lattice parameters calculated from the XRD data of Zn_1−*x*_Fe_*x*_O (*x* = 0.03 ÷ 0.15) and mean crystalline size (Debye-Scherrer formula [18]).

Zn_1−*x*_Fe_*x*_O (*x* = 0.03 ÷ 0.15)				
*x* = 0	*x* = 0.03	*x* = 0.05	*x* = 0.10	*x* = 0.15
$\left[\overset{´}{Å}\right]$ *a* = *b* = 3.249	*a* = *b* = 3.246	*a* = *b* = 3.250	*a* = *b* = 3.248	*a* = *b* = 3.251
$\left[\overset{´}{Å}\right]$ *c* = 5.206	*c* = 5.205	*c* = 5.205	*c* = 5.201	*c* = 5.206
$\left[{\overset{´}{Å}}^{3}\right]$ *V* = 47.590	*V* = 47.493	*V* = 47.435	*V* = 47.515	*V* = 47.535

[nm] average size	*D* = 37.9	*D* = 28.7	*D* = 26.2	*D* = 20.4
